# End-of-neoadjuvant treatment circulating microRNAs and HER2-positive breast cancer patient prognosis: An exploratory analysis from NeoALTTO

**DOI:** 10.3389/fonc.2022.1028825

**Published:** 2023-01-31

**Authors:** Serena Di Cosimo, Chiara M. Ciniselli, Sara Pizzamiglio, Vera Cappelletti, Marco Silvestri, Sarra El-Abed, Miguel Izquierdo, Mohammed Bajji, Paolo Nuciforo, Jens Huober, David Cameron, Stephen Chia, Henry L. Gomez, Marilena V. Iorio, Andrea Vingiani, Giancarlo Pruneri, Paolo Verderio

**Affiliations:** ^1^ Department of Advanced Diagnostics, Fondazione IRCCS Istituto Nazionale dei Tumori, Milan, Italy; ^2^ Unit of Bioinformatics and Biostatistics, Fondazione IRCCS Istituto Nazionale dei Tumori, Milan, Italy; ^3^ Breast International Group, Brussels, Belgium; ^4^ Novartis Pharmaceutical, Basel, Switzerland; ^5^ Institut Jules Bordet and l’Université Libre de Bruxelles (U.LB), Bruxelles, Belgium; ^6^ Molecular Oncology Group, Vall d’Hebron Institute of Oncology (VHIO), Barcelona, Spain; ^7^ Breast Center, University of Ulm, Ulm, Germany; ^8^ Breast Center, Cantonal Hospital St.Gallen, St. Gallen, Switzerland; ^9^ University of Leeds, Leeds, United Kingdom; ^10^ University of British Columbia, Vancouver, BC, Canada; ^11^ Department of Medical Oncology, Instituto Nacional de Enfermedades Neoplásicas, Lima, Peru; ^12^ Department of Medical Oncology, Universidad Ricardo Palma, Lima, Peru; ^13^ Department of Experimental Oncology, Fondazione IRCCS Istituto Nazionale dei Tumori, Milan, Italy

**Keywords:** circulating microRNA, HER2-positive breast cancer, prognosis, MiR-194-5p, neoadjuvant treatment

## Abstract

**Background:**

The absence of breast cancer cells in surgical specimens, *i.e.*, pathological complete response (pCR), is widely recognized as a favorable prognostic factor after neoadjuvant therapy. In contrast, the presence of disease at surgery characterizes a prognostically heterogeneous group of patients. Here, we challenged circulating microRNAs (miRNAs) at the end of neoadjuvant therapy as potential prognostic biomarkers in the NeoALTTO study.

**Methods:**

Patients treated within the trastuzumab arm (*i.e.*, pre-operative weekly trastuzumab for 6 weeks followed by the addition of weekly paclitaxel for 12 weeks; post-operative FEC for 3 cycles followed by trastuzumab up to complete 1 year of treatment) were randomized into a training (*n*= 54) and testing (*n*= 72) set. RT-PCR-based high-throughput miRNA profile was performed on plasma samples collected at the end of neoadjuvant treatment of both sets. After normalization, circulating miRNAs associated with event free survival (EFS) were identified by univariate and multivariate Cox regression model.

**Results:**

Starting from 23 circulating miRNAs associated with EFS in the training set, we generated a 3-circulating miRNA prognostic signature consisting of miR-185-5p, miR-146a-5p, miR-22-3p, which was confirmed in the testing set. The 3-circulating miRNA signature showed a C-statistic of 0.62 (95% confidence interval [95%CI] 0.53-0.71) in the entire study cohort. By resorting to a multivariate Cox regression model we found a statistical significant interaction between the expression values of miR-194-5p and pCR status (p.interaction =0.005) with an estimate Hazard Ratio (HR) of 1.83 (95%CI 1.14- 2.95) in patients with pCR, and 0.87 (95%CI 0.69-1.10) in those without pCR. Notably, the model including this interaction along with the abovementioned 3-circulating miRNA signature provided the highest discriminatory capability with a C-statistic of 0.67 (95%CI 0.58-0.76).

**Conclusions:**

Circulating miRNAs are informative to identify patients with different prognosis among those with heterogeneous response after trastuzumab-based neoadjuvant treatment, and may be an exploitable tool to select candidates for salvage adjuvant therapy.

## Introduction

Neoadjuvant therapy is progressively replacing adjuvant therapy and is emerging as a new standard of care for early-stage HER2-positive and triple negative breast cancer (1). Primarily used to downstage locally advanced tumors (2), neoadjuvant therapy has the potential to enable breast cancer operability as well as to increase conservative surgery and to reduce the extent of axillary node dissection (3). Furthermore, individual response to neoadjuvant therapy provides prognostic information and assists treatment decisions after surgery in patients with human epidermal growth factor receptor 2 (HER2)-positive and triple-negative breast cancer. Indeed, while patients with a pathological complete response (pCR), defined by the absence of invasive tumor in excised breast tissue and nodes, have a favorable prognosis, those with persistent disease within the surgical specimens require adjuvant therapy escalation (4, 5).

The efficacy of additional treatments in HER2-positive breast cancer patients not achieving a pCR after neoadjuvant therapy is being investigated in several studies (6), and has been reported as beneficial by the KATHERINE trial (7). Noteworthy, not all patients with an incomplete tumor response to neoadjuvant therapy relapse - two third of them are indeed disease-free at five years from surgery (8) - while up to 20% of patients who achieve a pCR eventually recur (9). This creates uncertainty, and calls into question the prognostic relevance of pCR. However, a reliable tool to separate patients at risk of relapse from those already cured after neoadjuvant therapy is currently lacking, hindering appropriate selection of adjuvant therapy escalation candidates. Primary tumor gene expression, proteomics and mutational profiling represent promising biomarkers, but need repeated tissue sampling and high-profile technology which limit their use in daily practice (10, 11). A possible alternative is offered by the development of a non-invasive procedure such as liquid biopsy. In particular, circulating microRNAs (miRNAs) are promising biomarkers due to their storage stability, easy handling, and promising expression signatures associated with treatment response (12). Baseline levels of circulating miRNA-21, -4734 and -150-5p (13, 14), or “on treatment” levels of circulating miRNA-140-5p (15) have already been associated with treatment response to HER2-targeted therapies. As it is common practice to offer post-operative treatment to non-responding patients, and pCR is not always reliable, we aimed to challenge circulating miRNAs detected at the end of neoadjuvant therapy as prognostic biomarkers. To this end, and given that most patients with HER2-positive early-stage breast cancer, in the face of different strategies to increase or decrease systemic therapy, continue to receive only chemotherapy and trastuzumab, we decided to analyze the association between pre-operative circulating miRNAs and event-free survival (EFS) of patients treated in the NeoALTTO trial (16) with trastuzumab-based therapy.

## Materials and methods

### Patients

This is an exploratory analysis of the multicenter phase III NeoALTTO study (16), which randomized patients with HER2-positive primary breast cancer >2 cm to lapatinib (*n* = 154), trastuzumab (*n*= 149), or their combination (*n*= 152) for 6 weeks, followed by paclitaxel for 12 weeks. Surgery was performed within four weeks from the last paclitaxel dose. After surgery, patients received fluorouracil, epirubicin, and cyclophosphamide for 3 cycles and continued the same anti-HER2-targeted agent of the neoadjuvant phase to complete 52 weeks of treatment. NeoALTTO primary endpoint was pCR; secondary endpoints included EFS, defined as the time from randomization to first event. As reported (15, 16), all enrolled patients signed the main study consent form, which included a non-specific clause for using blood samples collected at baseline, during treatment, prior to surgery, and eventually at the time of relapse for future research. The current analysis was approved (INT 186-13) by the Ethics Committee of Fondazione IRCCS Istituto Nazionale dei Tumori, Milano.

### Sample collection

Patients randomized to trastuzumab arm and with an available plasma sample collected prior to surgery were considered suitable for the purpose of this study, designed to address the prognostic value of end-of-neoadjuvant treatment circulating miRNAs. A training-testing approach was used for model building and confirmation, respectively.

### Circulating miRNA profiling data processing

Blood samples, collected in BDTM P-100 tubes (BD Bioscience), were separated within 2 hours of collection into plasma aliquots by centrifugation (2000-3000g for 15 minutes at room temperature) and stored at −80°C until assayed at the central biobank of Vall d’Hebron University Hospital (Barcelona, Spain). Plasma samples were shipped to Fondazione IRCCS Istituto Nazionale dei Tumori for RNA isolation as already reported (15). Briefly, reverse transcription and circulating miRNA profile was performed using the miRCURY LNA™Universal RT microRNA PCR system according to the Exiqon manufacturer’s instructions. A total of 752 circulating miRNAs were profiled using microRNA Ready-to-Use PCR, Human panel I+II in each sample. The amplification curves were analyzed using the Roche LC software for determination of quantification cycle (Cq) values. Consistently with our previous report (15), we considered background filtered (BF) Cq data as processed by Exiqon (i.e., for assays that do not yield any signal on the negative control, the upper limit of detection was set to Cq = 37; otherwise, it was set to 3 Cq lower than the Cq value of a negative control, 17). The BF Cq values were then processed to calculate the relative quantity (RQ) of each miRNA by using the comparative threshold cycle method (18) following the formula 2^-ΔCq^ (where ΔCq=Cq_miRNA_ - Cq_reference_). The Cq_reference_ was computed according to the overall mean approach (19).

### Statistical analysis

In the training set the association between circulating miRNAs levels (RQ considered on log2 scale) and event free survival (EFS) was assessed by resorting to a univariate Cox regression model. Event-free survival was defined as time from randomization to first event (i.e., events were defined as breast cancer relapse after surgery, second primary malignancy, death or failure to complete neoadjuvant therapy because of disease progression) (8). The relationship between each miRNA and clinical outcome was investigated by restricted cubic splines (20). In this selection step, according to the number of event per variable (EPV) (21), we considered as potentially relevant only those miRNAs detected in at least 10 patients experiencing the event of interest and in at least 85% of both training and testing sets (i.e. selected circulating miRNAs). Statistical significant circulating miRNAs at univariate analysis were included in multivariate Cox regression models following the all-subset approach (22) using penalized maximum likelihood estimations according to Firth method (23). For each model, the C-statistic (and its 95% Confidence Interval [CI]) computed according to Uno et al. (24) was used as pivotal measure for performance evaluation. Models with a statistically significant performance (i.e., with the lower 95%CI of the C-statistic >0.50) in the training set were then evaluated in the testing set. Those retaining a statistically significant performance in the testing set and including miRNAs with the same Hazard Ratio [HR] direction in both training and testing set were defined as promising prognostic signature(s). Noteworthy, models including miRNAs indicated in literature as haemolysis related (i.e., miR-16, miR-92a, miR-451 and miR-486) (25) were excluded as well as those with redundant circulating miRNAs according to the 95%CI of the Spearman correlation coefficient (i.e. upper limit of the 95%CI > 0.80 in absolute value). The “best” prognostic signature was eventually identified as that showing the highest prognostic performance in both the training and testing sets (**Supplementary Figure S1**). Next, by considering the whole study cohort (i.e., training and testing sets together), the prognostic performance of the “best” prognostic signature was evaluated with respect to clinico-pathological variables, i.e., estrogen receptor (ER) status (negative versus [vs] positive), nodal status (≥N1 vs N0), tumor size (>5cm vs ≤5cm), age (≥50 vs <50) and pCR (yes vs no). A 30-week landmark analysis was performed when pCR was considered as a covariate. Finally, a Cox regression model including each selected circulating miRNA the pCR status (main effects) together with their first order interaction term was implemented to highlight circulating miRNAs differently associated with EFS depending on pCR status. All statistical analyses were carried out with the SAS (version 9.4.; SAS Institute, Inc., Cary, NC) and R software (version 3.6.0; R Foundation for Statistical Computing) by adopting a significance alpha level of 5%. Prediction of target site of circulating miRNA(s) of interest was performed using miRWalk 3.0 (26). Functional enrichment of circulating miRNA targeted genes for Gene Ontology (GO) biological process terms and KEGG pathways was performed using the ClusterProfiler Bioconductor package, and a false discovery rate (FDR)< 0.05.

## Results

### Patient and tumor characteristics

Out of 149 patients treated within the NeoALTTO trastuzumab arm, 126 (85%) had evaluable circulating miRNA profile at the time of surgery (study cohort). The median age at breast cancer diagnosis was 48 years (interquartile range, 43-57). Most of the patients had clinical T2 (64%) and ≥N1 tumors (72%). Almost half (47%) had ER-positive tumors. pCR was observed in 31% of cases; a total of 40 events were reported at a median follow-up of 6.7 years (interquartile range, 6.1-6.9 years). Patients of the study cohort were randomized in a training (n= 54) and testing set (n= 72). No difference in clinico-pathological characteristics was observed between training and testing sets (**Table 1**), which were similar to the entire study cohort (**Supplementary Table S1**).

**Table 1:** Clinico-pathological features of the training and testing sets.

Age	Traning set n= 54		Testing set n= 72	
	n	*%*	n	*%*
<50 years	28	*52*	39	*54*
≥50 years	26	*48*	33	*46*
ER status				
Negative	27	*50*	40	*56*
Positive	27	*50*	32	*44*
Nodal status				
N0	15	*28*	20	*28*
≥N1	39	*72*	52	*72*
Tumor size				
≤5 cm	35	*65*	46	*64*
>5 cm	19	*35*	26	*36*
pCR				
No	37	*69*	50	*69*
Yes	17	*31*	22	*31*
**#Event**	17	*31*	23	*32*

ER, estrogen receptor; N, clinical nodal status at baseline; pCR, pathological Complete Response; event (i.e. breast cancer relapse after surgery, second primary malignancy, patient death or failure to complete neoadjuvant therapy because of disease progression).

### End-of-treatment circulating miRNA signature associated with EFS

In the training set, 23 circulating miRNAs were significantly associated with EFS by univariate analysis (**Supplementary Table S2**). For all these circulating miRNAs, a linear relationship between the EFS probability and their expression was found to be appropriate. By combining these 23 miRNAs into multivariate models following all-subset analysis approach (22), a total of 4 promising prognostic signatures were identified (**Table 2**). Among these 4 signatures, the model including miR-185-5p, miR-146a-5p, miR-22-3p, was selected as the “best” one. By using the regression coefficient of this model fitted on the whole study cohort, the 3-circulating miRNA signature was generated as following: (-0.062*miR-185-5p expression) + (-0.274*miR-146a-5p expression)+(0.105*miR-22-3p expression). **Supplementary Figure 2** reports the EFS probability pattern of the 3-circulating miRNA signature.The results of multivariate Cox regression model including these circulating miRNAs are reported in **Supplementary Table S3**. Notably in the study cohort, the C-statistic of the 3-circulating miRNA signature was 0.62 (95%CI 0.53-0.71), against a C-statistic of 0.58 (95%CI 0.48-0.67) of the model including ER expression, nodal status, tumor size, age and pCR.

**Table 2:** Prognostic performance of the promising models.

Promising models	Training set*	Testing set*
	C-statistic (95% CI)	C-statistic (95% CI)
miR-185-5p, miR-146a-5p, miR-22-3p	0.705 (0.517; 0.894)	0.700 (0.519; 0.880)
miR-146a-5p, miR-15b-3p, miR-22-3p	0.688 (0.551; 0.825)	0.698 (0.534; 0.861)
miR-24-3p, miR-15b-3p, miR-22-3p	0.681 (0.541; 0.820)	0.697 (0.512; 0.882)
miR-185-5p, miR-23a-3p, miR-22-3p	0.683 (0.509; 0.857)	0.672 (0.520; 0.824)

*A penalized Cox regression model was implements according to the EPV in the training and testing set.

### Circulating miRNA signature and clinico-pathological variables

**Figure 1:**
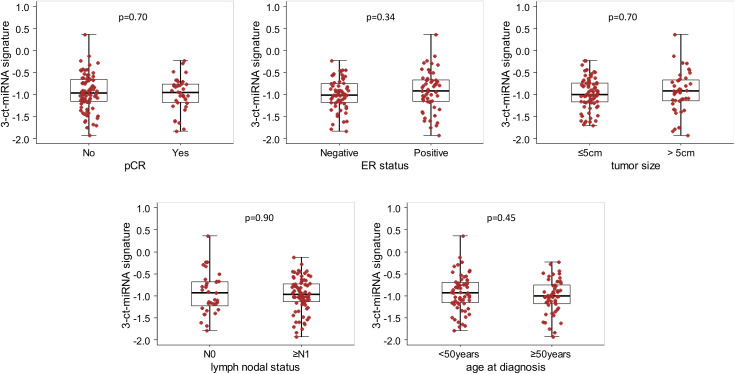
Box Plot of the 3-circulating miRNA signature according to clinical variables in the whole study cohort. Distribution of the 3-circulating miRNA signature expression levels according to pathological complete response (pCR), estrogen receptor (ER) status, primary tumor size, nodal status and age at diagnosis. Each box indicates the 25^th^ and 75^th^ percentiles. The horizontal lines inside the box indicate the median, and whiskers indicate the extreme measured values; individual value of the signatures are represented by dots. p=p.value of the Wilcoxon test.

Noteworthy, this 3-circulating miRNA signature was evenly distributed among clinico-pathological variables (**Figure 1**). Moreover, the 3-circulating miRNA signature retained its prognostic performance with respect to EFS even after adjusting for each of the considered clinico-pathological variables (**Supplementary Table S4**).

### Prognostic value of circulating miRNAs by pCR status

**Figure 2:**
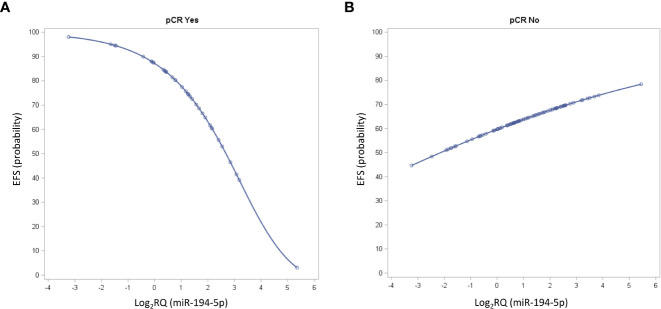
Seven-year Event Free Survival (EFS) probability curves for circulating miR-194-5p values according to pathological complete response (pCR) status. The curve depicts the predicted EFS probability of the circulating miR-194-5p expression levels considered on its continuous scale in patients **(A)** with pCR and **(B)** without pCR.

As pCR is a driver of EFS in the NeoALTTO and other neoadjuvant studies, we next analyzed the prognostic value of miRNA according to pCR status. For this purpose, the expression levels of each of the 132 selected miRNAs detected in the study cohort was evaluated thought a multivariate Cox regression model with pCR status and the first-order interaction between miRNA and pCR. We found circulating miR-194-5p with a statistically significant interaction term at alpha level of 0.01 (p.interaction =0.005). The HR estimate in patients with pCR was 1.83 (95%CI 1.14-2.95), and 0.87 (95%CI 0.69-1.10) in those without pCR. **Figure 2** reports the EFS probability plot for circulating miR-194-5p expression levels (Log2 RQ) according to pCR status.

### End-of-treatment microRNA added prognostic value

For exploratory purposes, an analysis was performed by adding the interaction terms together with the corresponding main effects (circulating miR-194-5p and pCR) to the 3-circulating miRNA signature. The highest prognostic performance in terms of C-statistics was observed for the complete multivariate model (**Table 3**).

**Table 3:** Prognostic performance of multivariate Cox regression models in the study cohort.

Model	C-statistic	95% CI	
3-circulating miRNA signature + pCR (Yes *vs* No)^a^	0.62	0.53	0.71
miR-194-5p + pCR+ (miR-194-5p*pCR)^a^	0.62	0.54	0.71
3-circulating miRNA signature + pCR + miR-194-5p + (miR-194-5p*pCR)^a^	0.67	0.58	0.76

a 30-week landmark analysis was performed when pCR was considered in the multivariate Cox regression model; CI, confidence interval.

## Discussion

Not all early-stage breast cancer patients with residual disease after neoadjuvant therapy have a poor prognosis; on the other hand, some patients achieving a pCR eventually relapse. Therefore, biomarkers are needed to properly identify patients at risk who are ideal candidates for additional post-surgical therapies. Using high-throughput analysis of plasma samples collected within a prospective randomized trial (16), we herein reported the first study investigating circulating microRNAs extensively, and not according to a pre-specified candidate panel, at the end of neoadjuvant therapy, and in association with prognosis. Several key findings with biological relevance and clinical potential were identified.

Firstly, we identified a circulating signature composed of miR-185-5p, miR-146a-5p and miR-22-3p able to discriminate among patients treated with trastuzumab-based therapy with different prognosis. The functions of miRNAs composing our prognostic signature have been associated with tumor-related processes (proliferation, apoptosis, migration/invasion), and response to treatment. Specifically, miR-185-5p seems to act as a tumor suppressor in cancer progression and spreading, at least in central nervous system (27), and gastrointestinal malignancies (28). The precise effects and detailed mechanisms of miR-185-5p in breast cancer are yet to be defined. However, a recently reported pre-clinical study shows that over-expression of miR-185-5p is associated with reduced chemosensitivity (29). Iorio et al. first reported that miR145-5p acts as a tumor suppressor in a variety of tumors, including breast cancer (30); subsequently, miR-145-5p was shown to modulate immune response by targeting the 3’-untranslated region of Toll-like receptor 4 (31), and to increase epithelial-mesenchymal transition through the control of N-cadherin, vimentin and E-cadherin protein expression levels (32). Finally, miR-22 was found both as a tumor suppressor and a promoter in previous studies (33, 34). However, its serum expression levels have already been associated with poor prognosis in breast cancer patients (35). Consistent with these literature data, our analysis showed that both miR-185-5p and miR-146a-5p have a protective prognostic effect, as opposed to miR-22. Furthermore, an integrated analysis of miRNA target gene networks drew much attention because there are many common signaling pathways modulated by differentially expressed mRNAs and shared through GO and KEGG analysis (**Supplementary Figure S3**). Although circulating miRNAs are not necessarily expressed at the tumor tissue level, it is worthy to note that among the enriched terms, those related to growth factor signaling, metastatic spreading processes and immune response were shared by all dysregulated circulating miRNAs after trastuzumab treatment. These findings are intriguing because development of predictors of recurrence after neoadjuvant therapy is still in its infancy, and presently the only established prognostic factors are stage and hormone receptor status (36). An attempt has been made with TILs (37). However, TILs assessment may be difficult after neoadjuvant therapy (38), so that Asano et al. proposed to combine their evaluation with residual cancer burden, which takes into account tumor dimension, cellularity of tumor bed, and axillary nodal burden (39). In addition TILs data in HER2-positive breast cancer are controversial (40), with several studies suggesting that high TILs values in residual disease after neoadjuvant therapy are associated with worse rather than improved prognosis (reviewed in 37).

Secondly, the 3 circulating miRNA signature ensures a C-statistic of 0.62 (0.53-0.7), instead of 0.58 (0.48-0.67) of the model including clinico-pathological variables, age, stage, estrogen receptor status and pCR. Thus, only three circulating markers assessed at a single time point offer a similar if not superior discriminatory capability of different variables assessed on patient, and primary tumor at baseline or after treatment. Furthermore, the discriminatory capability of the 3-circulating miRNA signature remained significant when clinico-pathological variables were included in multivariate analysis. All these findings support the development of our 3 circulating miRNA signature as a parsimonious and independent prognostic tool.

Thirdly, despite the favorable prognostic effect of invasive disease eradication, a few of patients with pCR eventually relapse (9, 10). Tumor recurrences are in the range of 10-15% at 5 years from surgery, as reported in the large pooled analysis of German Breast Group neoadjuvant studies on 2188 patients (41), and from a recent meta-analysis on 5748 patients (42). These findings are counterintuitive since complete eradication of cancer cells in breast and axilla has been proposed as a maximum effect reflecting the eradication of micrometastatic disease with no room for improvement. Yet, risk of relapse in patients with pCR increases with advanced stage at initial diagnosis, HER2-overexpression, younger age, and premenopausal status. Besides, patients who obtain pCR with the addition of trastuzumab have a better prognosis than patients who obtain pCR with chemotherapy alone (43). Recently, accumulating data suggest that patients who achieve pCR with HER2-dual blockade have a better prognosis than those who achieve pCR with single anti-HER2 agent (44). Although we cannot exclude that these results are due to the imbalance in the size of the subgroups, with fewer patients in the control arm achieving a pCR, these data challenge the idea that all pathological responses born equal and reinforce that of pCR quality. In this context, our data demonstrate for the first time that increased levels of circulating -miR-194--5p are associated with dismal prognosis exclusively in patients achieving a pCR following trastuzumab. miR-194-5p is a p53-responsive miRNA capable of inducing cell cycle arrest and inhibiting cell proliferation, migration, invasion and colony formation (45). These data are inconsistent with our results. However, it has recently been reported that the function miR-194 turns tumorigenic when treatment-sensitive cells promote stem cell survival during the so called “dying for surviving” phenomenon (46). Specifically, miR-194-5p is contained in the exosomes of dying cells and is released to residual ALDH- positive tumor-repopulating cells for their recovery. This finding is consistent with the enrichment of cells with stemness phenotype in occult metastatic lesion (47), and the overexpression of stemness signatures in triple negative breast cancer primary tumor of patients who eventually relapse regardless a pCR (10).

This study has some limitations. First, due to its retrospective nature, biological data were available in most but not all patients included in the NeoALTTO trastuzumab arm. Secondly, the analyzed sample size and the number of breast cancer events prevented sub-group analysis. Finally, the number of patients included in the analysis limits the impact of our findings. Therefore, the results of this study need a further confirmation in a much larger patient population, and with an extended follow-up period. Lastly, the prognostic value of TILs was not evaluated in this study. This would be interesting to be assessed in future studies. In addition, studied patients received additional adjuvant chemo-, ± endocrine- and trastuzumab-therapy and we are unaware of the impact of these treatments on miRNAs and *vice versa*. In view of developing a clinically usable assay, the identification of a limited number of reference miRNAs as well as assay-oriented step(s) should be considered, as described by Verderio et al. (22). As regards the selection of reference miRNAs, they should be properly chosen to resemble the overall mean (19), for example using the procedure we developed (48). The definition of operative procedures for miRs processing and detection as well as the evaluation of the assay performance and robustness, together with the clinical interpretability represent key aspects that should be opportunely addreesd during the assay-oriented step(s).

In conclusion, we have identified a 3 circulating miRNA signature able to differentiate among patients with distinct prognosis after trastuzumab-based neoadjuvant therapy, and the unique circulating-miR-194-5p associated with recurrence after pCR attainment, which warrant further investigation in additional studies. If confirmed, these miRNAs and their possible mechanisms of action could aid the development of new post-neoadjuvant strategies for high risk breast cancer patients. Furthermore, given the absence of tumor tissue in patients attaining a pCR, our results reinforce liquid biopsy as a promising tool to quantify and analyze residual breast cancer burden beyond pathological findings and to predict tumor evolution for post-operative therapy personalization.

## Data availability statement

The raw data supporting the conclusions of this article will be made available by the authors upon reasonable request.

## Ethics statement

The studies involving human participants were reviewed and approved by Ethics Committee of Fondazione IRCCS Istituto Nazionale dei Tumori. The patients/participants provided their written informed consent to participate in this study.

## Author contributions

SDC conceived of the presented idea. SDC, CMC, SP and PV contributed to the design and implementation of the research, to the analysis of the results and to the writing of the manuscript. VC and MS verified the analytical methods. All authors contributed to the article and approved the submitted version.
