# Supplementary material for: End-of-neoadjuvant treatment circulating microRNAs and HER2-positive breast cancer patient prognosis: An exploratory analysis from NeoALTTO
Source: Front Oncol. 2023 Jan 31;12:1028825. doi: 10.3389/fonc.2022.1028825 (PMC9927225; doi:10.3389/fonc.2022.1028825)

Supplementary Table S1. Clinico-pathological features of the NeoALTTO trastuzumab arm and of current entire study cohort

	**NeoALTTO** Trastuzumab arm		**Study cohort**	
	**n=149**		**n=126**	
**Age**	**n**	**%**	**n**	**%**
<50 years	77	*52*	67	*53*
≥50 years	72	*48*	59	*47*
**ER status**				
Negative	80	*54*	67	*53*
Positive	69	*46*	59	*47*
**Nodal status**				
N0	108	*72*	91	*72*
≥N1	41	*28*	35	*28*
**Tumor size**				
≤5 cm	93	*62*	81	*64*
>5 cm	56	*38*	45	*36*
**pCR**				
No	105	*70*	87	*69*
Yes	44	*30*	39	*31*
**#Event**	45	*30*	40	*32*

ER: estrogen receptor; N: clinical nodal status at baseline; pCR: pathological Complete Response. event (i.e. breast cancer relapse after surgery, second primary malignancy, patient death or failure to complete neoadjuvant therapy because of disease progression).

Supplementary Table S2. Results of univariate Cox regression model of selected miRNAs in the training set

circulating miRNA	HR	95% CI		p-value
let-7d-3p	0.49	0.27	0.90	0.021
miR-130b-3p	0.60	0.39	0.94	0.027
miR-132-3p	0.45	0.23	0.87	0.018
miR-146a-5p	0.63	0.44	0.92	0.016
miR-15b-3p	0.66	0.46	0.96	0.030
miR-16-5p	0.69	0.48	0.98	0.039
miR-17-5p	0.54	0.34	0.88	0.013
miR-185-5p	0.54	0.34	0.87	0.010
miR-199a-3p	0.65	0.45	0.94	0.021
miR-223-3p	0.62	0.39	1.00	0.050
miR-22-3p	0.57	0.33	0.96	0.035
miR-23a-3p	0.49	0.26	0.93	0.030
miR-24-3p	0.56	0.36	0.88	0.012
miR-27b-3p	0.61	0.38	0.96	0.035
miR-30d-5p	0.56	0.36	0.87	0.010
miR-30e-3p	0.49	0.27	0.90	0.021
miR-30e-5p	0.57	0.36	0.90	0.016
miR-328-3p	0.68	0.49	0.95	0.025
miR-425-5p	0.60	0.39	0.94	0.025
miR-584-5p	0.64	0.41	0.99	0.045
miR-590-5p	0.69	0.48	0.99	0.046
miR-92a-3p	0.54	0.34	0.88	0.013
miR-93-5p	0.65	0.43	1.00	0.049

HR: Hazard Ratio; CI: confidence interval.

Supplementary Table S3. Three circulating miRNAs multivariate Cox regression model in the training and testing sets and in the whole study cohort

	Training set			Testing set			Study cohort		
**Model**	**HR**	**95% CI**		**HR**	**95% CI**		**HR**	**95% CI**	
miR-185-5p	0.67	0.38	1.17	0.88	0.63	1.25	0.94	0.71	1.25
miR-146a-5p	0.75	0.48	1.25	0.65	0.46	0.94	0.76	0.58	1.00
miR-22-3p	1.00	0.46	2.24	1.95	1.17	3.43	1.11	0.94	1.32

HR: Hazard Ratio; CI: confidence interval. In the training and testing cohort a penalized multivariate Cox regression was implemented.

Supplementary Table S4. Multivariate Cox regression models including the 3 circulating miRNAs and each of the considered clinico-pathological variables in the overall study cohort

Model	HR	95% CI		C-statistic (95%CI)
miR-185-5p	0.940	0.708	1.248	0.615 (0.525-0.705)
miR-146a-5p	0.761	0.576	1.004	
miR-22-3p	1.111	0.937	1.317	
miR-185-5p	0.929	0.706	1.226	0.619 (0.523-0.715)
miR-146a-5p	0.728	0.546	0.971	
miR-22-3p	1.135	0.958	1.352	
ER status (Neg vs Pos)	1.533	0.771	3.108	
miR-185-5p	0.941	0.707	1.253	0.619 (0.530-0.708)
miR-146a-5p	0.764	0.578	1.010	
miR-22-3p	1.107	0.937	1.315	
Tumor size (>5 vs ≤5)	1.150	0.573	2.236	
miR-185-5p	0.937	0.704	1.244	0.615 (0.526-0.704)
miR-146a-5p	0.761	0.575	1.007	
miR-22-3p	1.109	0.939	1.318	
Nodal status (≥N1vs N0)	0.936	0.474	1.937	
miR-185-5p	0.943	0.710	1.251	0.617 (0.528-0.706)
miR-146a-5p	0.770	0.583	1.016	
miR-22-3p	1.103	0.934	1.312	
Age (≥ 50 vs <50)	0.763	0.376	1.493	
miR-185-5pa	0.934	0.702	1.239	0.618 (0.526-0.710)
miR-146a-5pa	0.768	0.583	1.014	
miR-22-3pa	1.104	0.934	1.313	

pCR (Yes vs. No)[a]	0.731	0.329	1.487	

HR: Hazard Ratio; CI: confidence interval; ER: estrogen receptor; pCR: pathological complete response. [a]Landmark analysis was performed.

Supplementary Figure S1. Statistical analysis workflow.

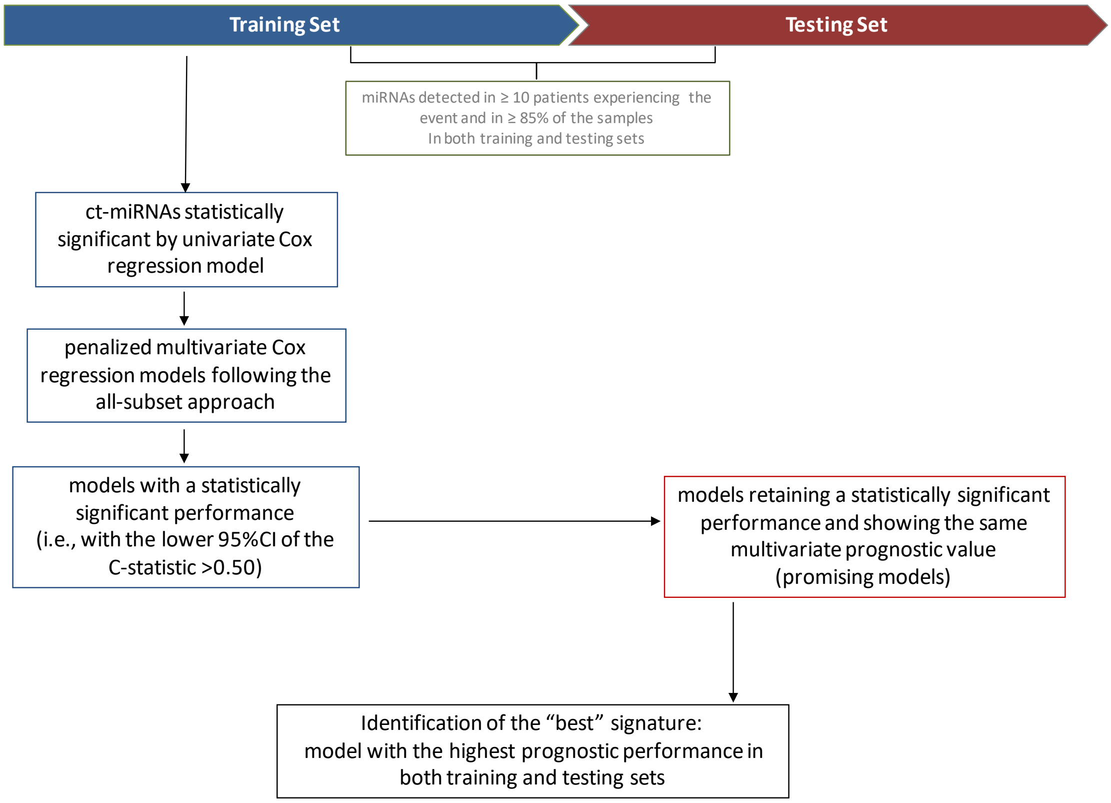

Supplementary Figure S2. Seven-years Event Free Survival (EFS) probability curves for the 3-circulating miRNA signature in the study cohort. The curve depicts the predicted EFS probability of the signature levels considered on its continuous scale in all patients .

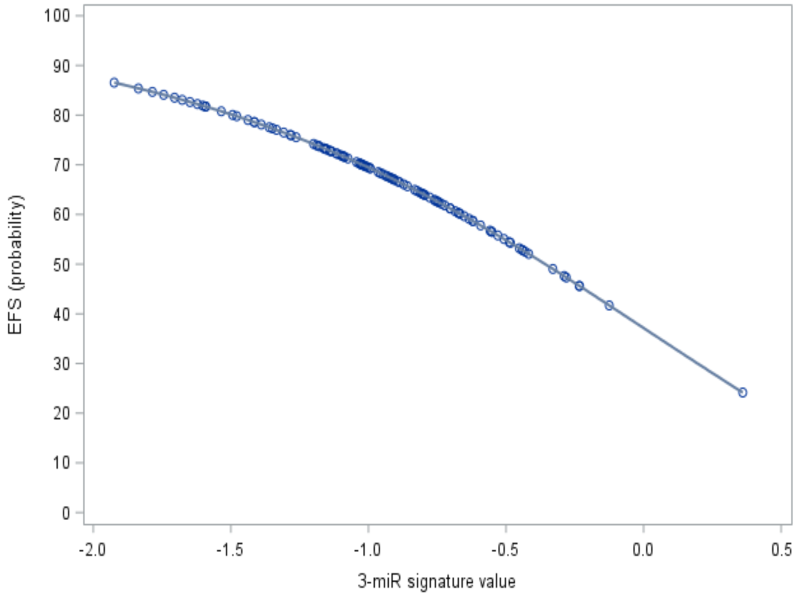

Supplementary Figure S3. Functional interactions network analysis of miR-185-5p, miR-146a-5p and miR-22-3p. Specific (represented in blue) and common targeted signaling pathways (represented in yellow) of circulating miRNAs associated with EFS (i.e., miR-185-5p, miR-146a-5p and miR-22-3p, represented in red) obtained from GO and KEGG database analyses.

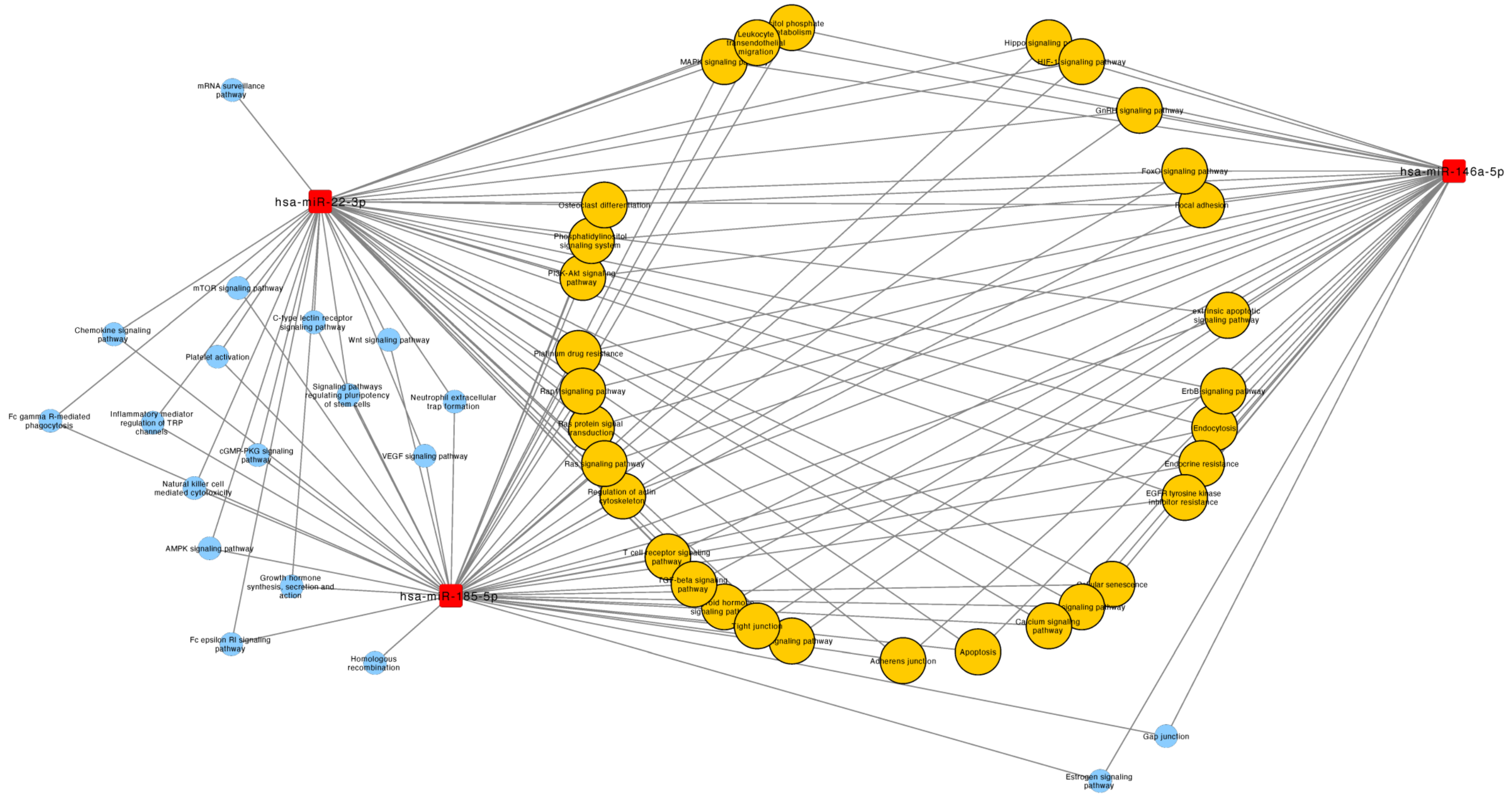

Supplement: Supplementary file 1 [file DataSheet_1.pdf]
